# Bilateral blindness from optic chiasm metastasis as the initial presentation of stage 4 neuroblastoma: A case report

**DOI:** 10.1097/MD.0000000000048061

**Published:** 2026-03-13

**Authors:** Heba Amer, Sultaneh Haddad, Saffaa Ahmed Hersi, Ismail Fawaz Al Malla, Valentina Obaid, Buseyna Dahik, Razan Abbas

**Affiliations:** a Faculty of Medicine, Damascus University, Damascus, Syrian Arab Republic; b Division of Pediatrics, Children’s University Hospital, Damascus, Syrian Arab Republic; c Faculty of Medicine, Ain Shams University, Cairo, Egypt; d Division of Dermatology, National Hospital of Latakia, Latakia, Syrian Arab Republic; e Faculty of Medicine, Al Hawash Private University, Homs, Syrian Arab Republic; f Faculty of Medicine, Ankara Yilidirim Beyazit University, Ankara, Turkey; g Faculty of Medicine, Latakia University, Latakia, Syrian Arab Republic.

**Keywords:** bilateral blindness, case report, intracranial extension, neuroblastoma, optic chiasm

## Abstract

**Rationale:**

Neuroblastoma is the most common extracranial solid tumor in early childhood and may present with nonspecific symptoms that delay diagnosis, particularly in resource-limited settings. Intracranial involvement affecting the optic pathway is exceptionally rare. This report describes an unusual presentation of neuroblastoma leading to irreversible bilateral blindness.

**Patient concerns:**

A 29-month-old male with albinism presented with fever, abdominal pain, and pallor and was initially treated for a presumed urinary tract infection. Within days, he developed awakening headache followed by sudden bilateral blindness.

**Diagnoses:**

Imaging studies, including ultrasound, computed tomography, and magnetic resonance imaging, revealed a large right suprarenal mass with widespread metastases involving the optic chiasm and cranial bones. Histopathological examination confirmed stage 4 neuroblastoma.

**Interventions:**

The patient underwent surgical debulking followed by chemotherapy according to the St. Jude high-risk protocol.

**Outcomes:**

Partial regression of metastatic lesions was achieved; however, visual loss remained permanent.

**Lessons:**

Neuroblastoma may present with atypical neurological manifestations, including optic chiasm metastasis leading to blindness. Early abdominal imaging in pediatric patients with unexplained systemic or abdominal symptoms is essential to prevent delayed diagnosis and irreversible complications.

## 1. Introduction

Neuroblastomas are the most prevalent extracranial tumors in children younger than 4 years, occurring at a rate of approximately 1 to 3 cases per 100,000.^[1]^ They constitute about 6% to 10% of all pediatric cancers and are responsible for nearly 15% of cancer-related mortality in children.^[1]^ It originates from neural crest cells at various sites within the sympathetic nervous system, with the abdomen being the most frequent location.^[2]^ The symptoms of neuroblastoma are influenced by its original site and the degree of metastasis, if present.^[3]^ The abdomen is the most frequent primary location, with potential spread to the bones, lymph nodes, liver, brain, eye sockets, lungs, and central nervous system.^[3]^ Neuroblastoma manifests with various clinical symptoms, including eye protrusion, bruising around the eyes, abdominal swelling, bone pain, reduced blood cell counts, fever, anemia, high blood pressure, paralysis, watery diarrhea, and skin nodules.^[3]^ Blindness has been reported in extremely rare instances, documented only in a few isolated cases.^[4]^ CT and MRI imaging are essential in the diagnosis of neuroblastoma, especially for precisely identifying the tumor’s site of origin.^[1]^ At present, chemotherapy continues to serve as the primary modality of treatment for this condition.^[1]^

In this study, we present a significant case of 29-month-old male child with metastatic neuroblastoma that led up to an irreversible bilateral blindness.

## 2. Case presentation

A 29-month-old male child, born at term following an uncomplicated pregnancy and delivery, with a known diagnosis of albinism, presented to the pediatric clinic with a 2-day history of abdominal pain, fever, and pallor. Initial laboratory work-up included a complete blood count, but no abdominal imaging was performed. The clinical picture led to a preliminary diagnosis of urinary tract infection and anemia.

Within a few days, the patient developed a severe awakening headache accompanied by the sudden onset of bilateral blindness. Ophthalmologic examination revealed mid-dilated, nonreactive pupils lacking fixation or tracking responses, along with grade 3 papilledema. Eyelids appeared normal.

**Figure 1.**
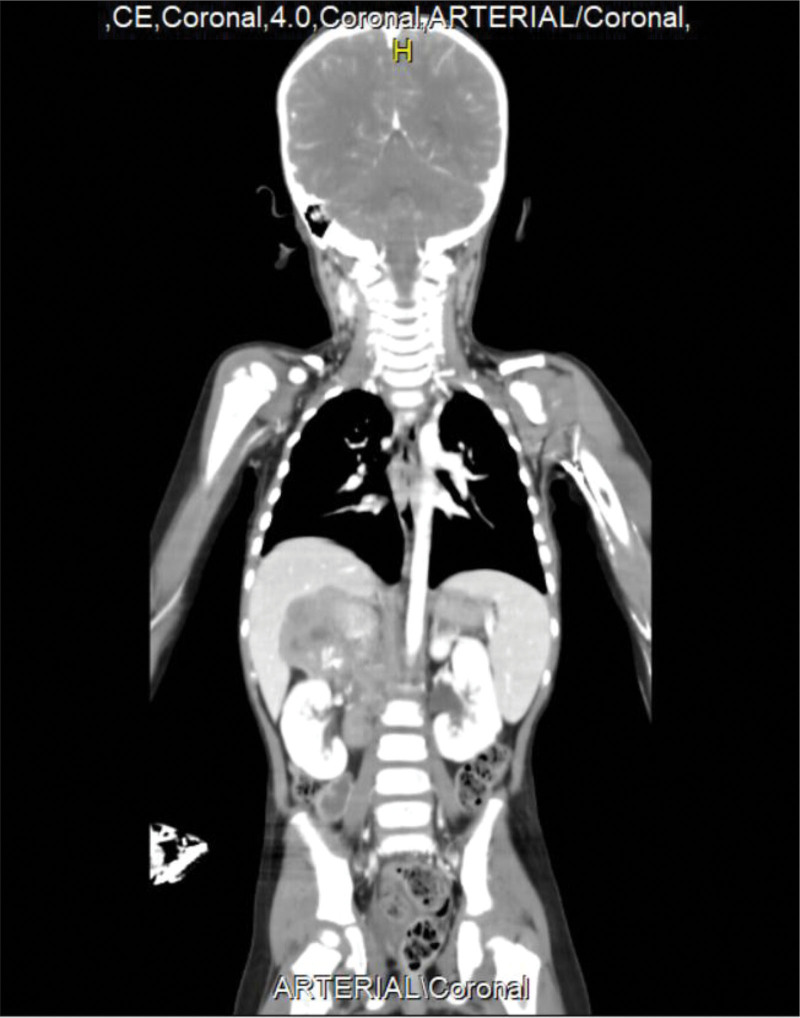
shows a contrast whole-body CT that illustrates a heterogenous calcified mass above the right kidney along with lymphadenopathies. CT = computed tomography.

Subsequent abdominal and pelvic ultrasonography revealed a heterogeneous suprarenal mass measuring 11 cm in diameter, located superior to the right kidney. Contrast-enhanced whole-body computed tomography (CT) confirmed a heterogeneous, calcified mass consistent with neuroblastoma, along with cervical, thoracic, and pelvic lymphadenopathy (Fig. 1).

**Figure 2.**
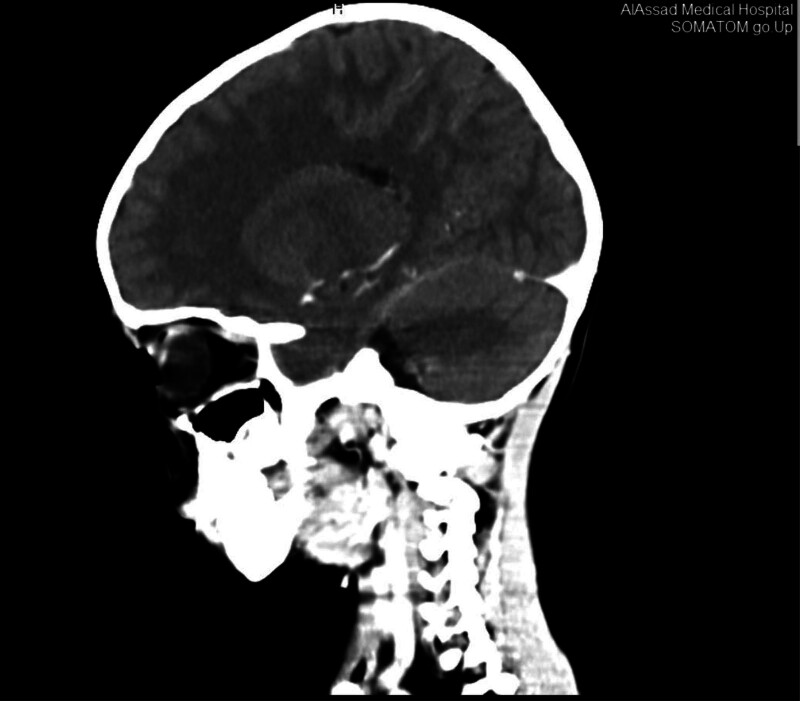
is a cranial CT that shows a lesion in the Sella turcica with extension into the optic chiasm and sphenoid bone. CT = computed tomography.

**Figure 3.**
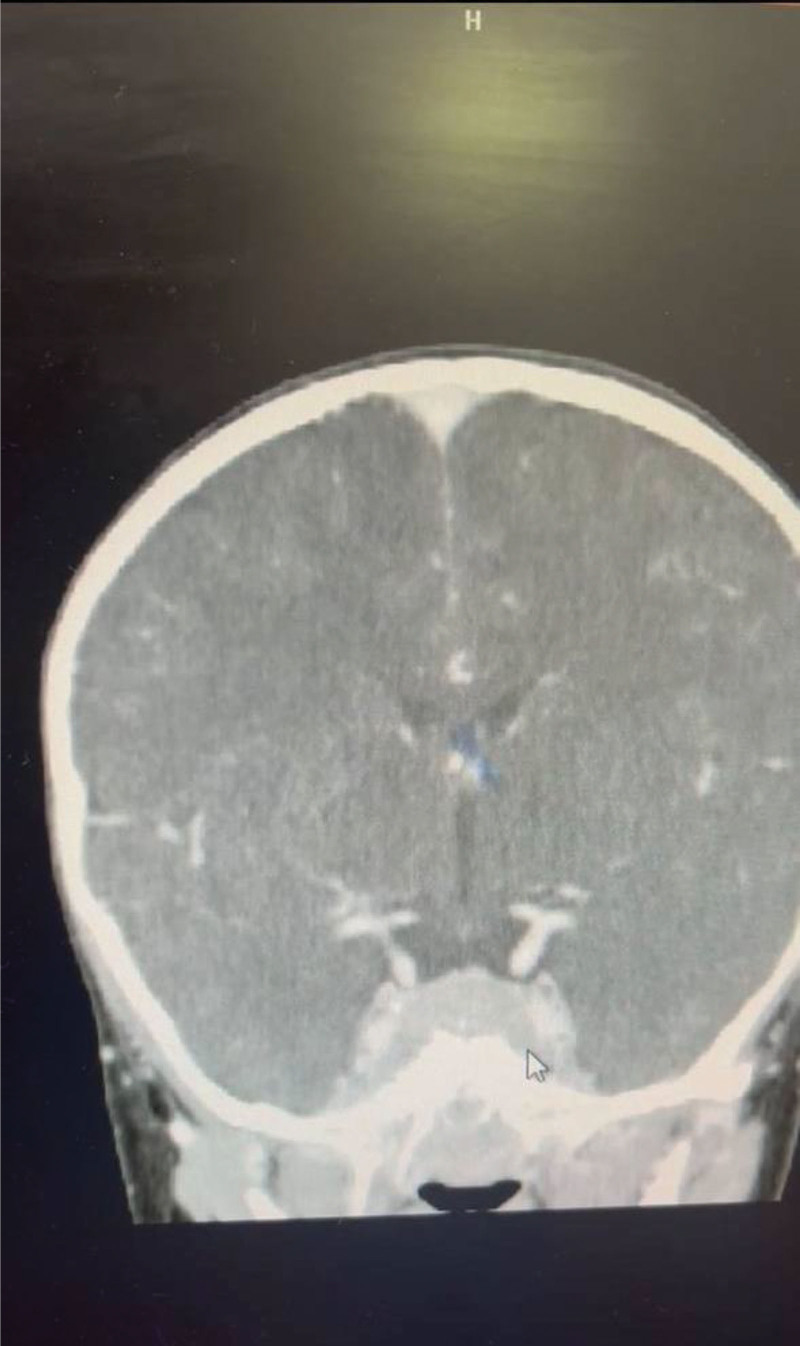
shows the infiltrates of the metastasis.

**Figure 4.**
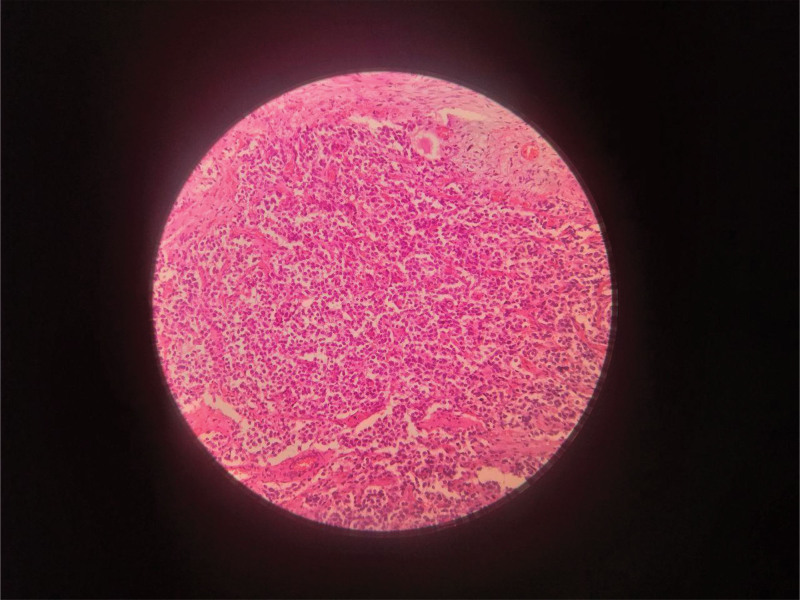
is a microscopic examination revealing a highly cellular neoplasm composed of small, round neuroblastic cells with hyperchromatic nuclei and scant cytoplasm, arranged in sheets within a fibrillary neuropil background.

**Figure 5.**
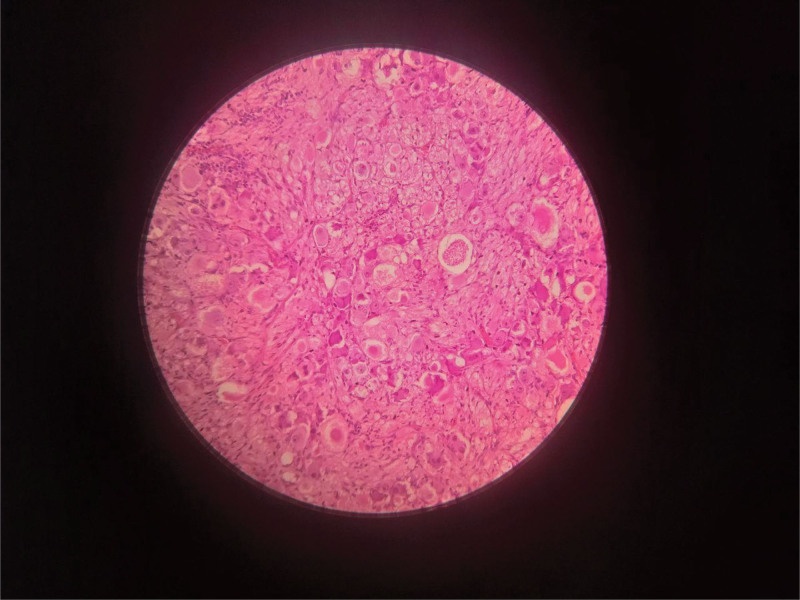
shows large ganglion-like cells with abundant eosinophilic cytoplasm and vesicular nuclei consistent with ganglioneuroblastic differentiation.

Cranial CT imaging demonstrated 2 intracranial lesions: the first situated in the Sella turcica with extension into the optic chiasm and sphenoid bone; the second infiltrating the ethmoid bone (Figs. 2 and 3). Magnetic resonance imaging (MRI), utilizing T2-weighted and FLAIR sequences, revealed multiple enhancing, high-density metastatic lesions.

histopathological analysis of the biopsy demonstrated hyperchromatic, small, round and blue cells (Fig. 4) some with ganglion differentiation arranged in rosette-like structures (Homer Wright rosettes) (Fig. 5), and numerous individually scattered malignant cells – findings consistent with a mature neuroblastoma stage 4.

**Figure 6.**
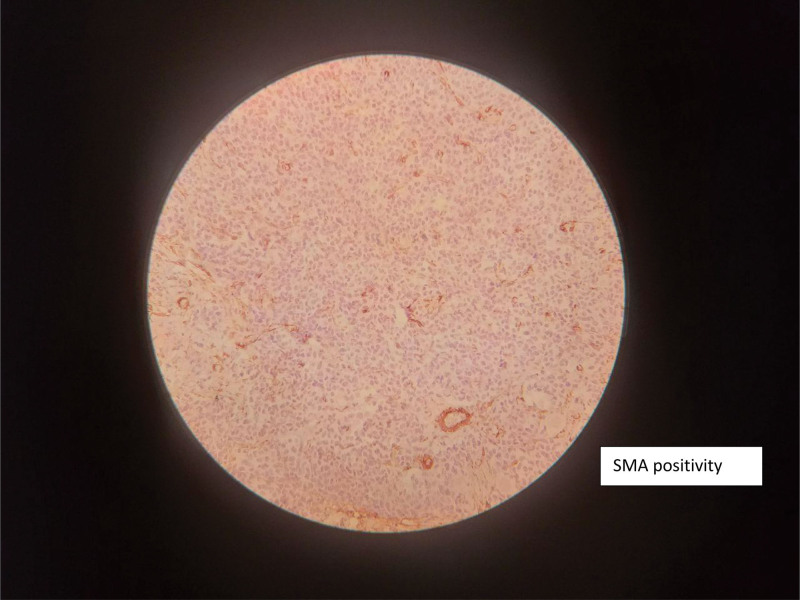
Immunohistochemical staining demonstrating positivity for SMA. SMA = smooth muscle actin.

**Figure 7.**
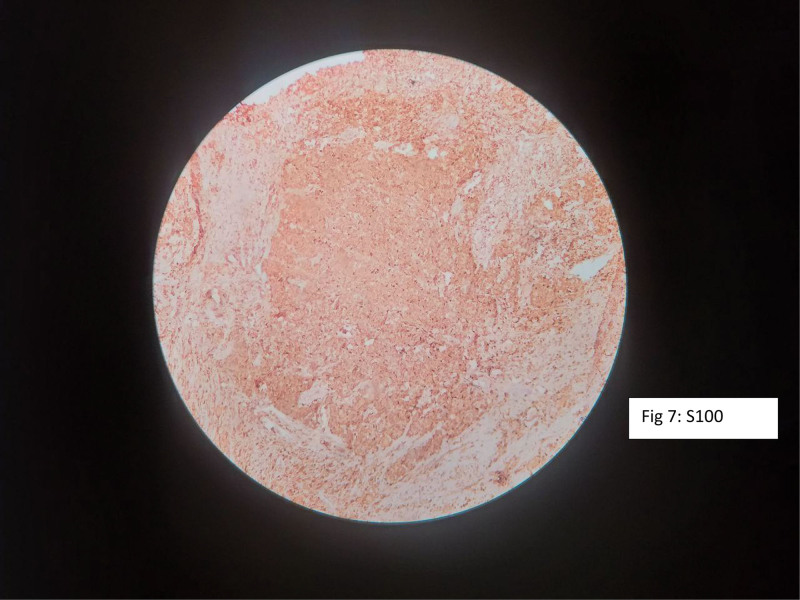
Immunohistochemical staining demonstrating positivity for S-100 protein.

**Figure 8.**
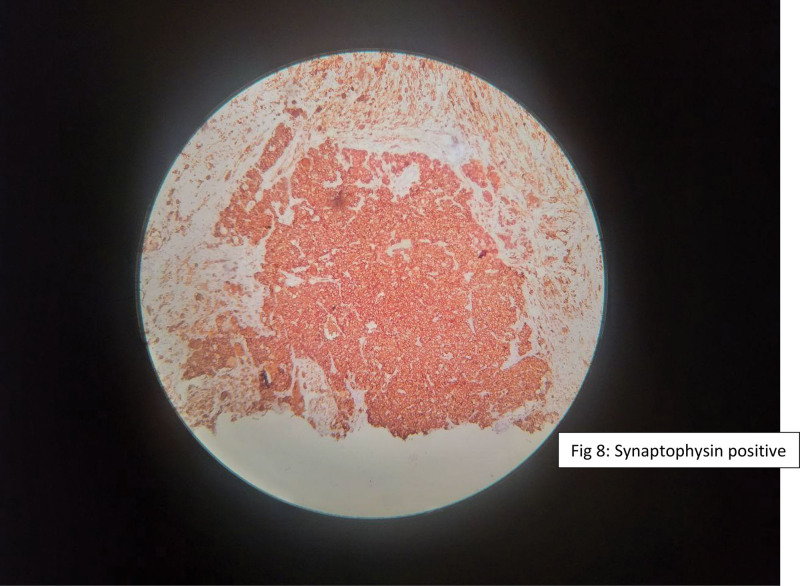
Immunohistochemical staining demonstrating positivity for synaptophysin.

**Figure 9.**
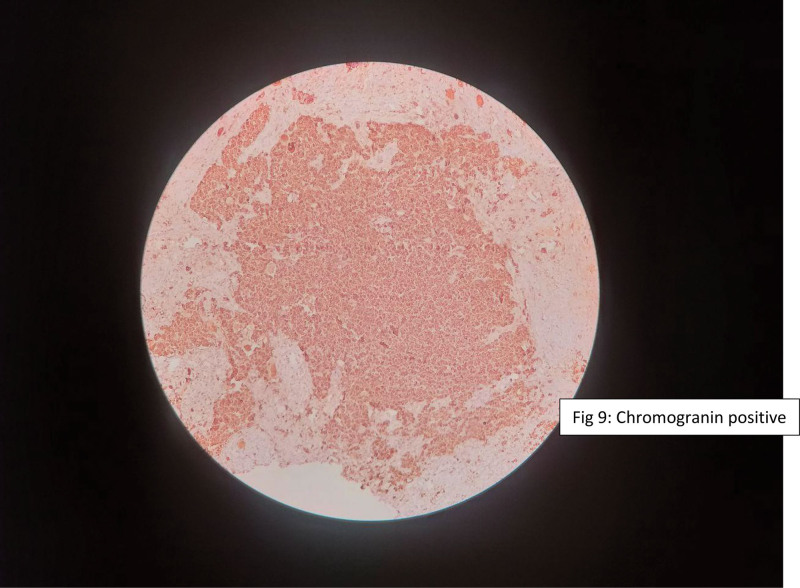
Immunohistochemical staining demonstrating positivity for chromogranin.

Immunohistochemical staining demonstrated tumor cell positivity for smooth muscle actin, S-100 protein, chromogranin, and synaptophysin, supporting the diagnosis of a neuroblastic tumor with both neuroendocrine and stromal differentiation (Figs. 6–9).

**Figure 10.**
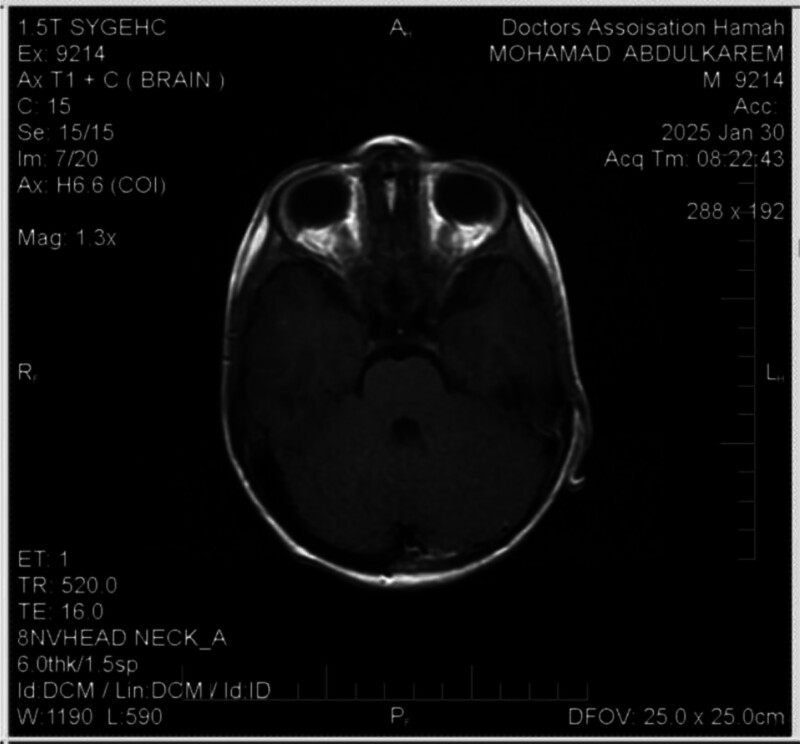
Is a follow-up MRI that shows resolution of intracranial metastasis. MRI = magnetic resonance imaging.

The patient was initiated on the St. Jude protocol for high-risk neuroblastoma. Surgical intervention achieved 90% resection of the primary mass. Chemotherapy was continued postoperatively, and follow-up MRI and CT imaging showed resolution of metastatic lesions (Fig. 10).

## 3. Outcomes

Following initiation of the St. Jude high-risk protocol and 90% surgical debulking of the primary adrenal mass, the patient demonstrated partial radiologic improvement. Follow-up CT and MRI showed regression of several metastatic lesions, decreased tumor burden, and stabilization of previously identified cranial lesions. Clinically, systemic symptoms such as fever and abdominal pain resolved with treatment. However, the patient’s visual loss remained irreversible due to extensive infiltration of the optic chiasm at presentation. Ongoing management includes continuation of chemotherapy, with plans for autologous stem-cell transplantation as consolidation therapy. Additional follow-up imaging could not be performed because of financial limitations. The patient remains under active oncologic care.

## 4. Ethical considerations

According to local regulations, formal ethical committee approval is not required for single-patient case reports. Written informed consent for publication of the patient’s clinical information and imaging was obtained directly from the patient’s parents, who serve as the legal guardians.

## 5. Discussion

Neuroblastoma is the most common extracranial solid tumor in children, accounting for 8% to 10% of pediatric cancers worldwide.^[5]^ The median age at diagnosis is 17 to 18 months, with over 90% of cases occurring before age 5 and a slight male predominance.^[5]^ Primary tumors most often arise in the abdomen, particularly the adrenal medulla (~50%), though they may occur anywhere along the sympathetic chain.^[6,7]^ Incidence varies by geography and income, with higher rates in high-income countries.^[5,8]^ Global trends demonstrate declining incidence and mortality in high-SDI regions, whereas a rising burden is observed in lower-SDI regions, partly due to limited healthcare resources and reduced access to early diagnostic services.^[5]^ In this context, our patient – a 29-month-old male from Syria – falls within the broader pediatric age distribution for neuroblastoma, highlighting that children beyond infancy remain at risk, particularly in resource-constrained settings.

Most neuroblastomas are sporadic, although familial cases have been linked to germline mutations, such as ALK and PHOX2B.^[9]^ Somatic alterations including MYCN amplification, 1p36 deletion, and 11q loss correlate with disease stage and prognosis.^[10]^

Neuroblastoma exhibits considerable clinical and biological variability, with disease manifestations and outcomes influenced by patient age, tumor stage, and primary site.^[7]^ Presentation may include localized gastrointestinal findings – such as abdominal mass, distension, or abdominal pain – or constitutional features including anorexia, weight loss, fever, failure to thrive, and pallor.^[11]^ In our patient, initial findings were fever, abdominal pain, and pallor. Complete blood count confirmed anemia, consistent with reports that a substantial proportion of children with neuroblastoma exhibit anemia, particularly those with high-risk or advanced disease.^[12,13]^ Opsoclonus–myoclonus-ataxia syndrome, a rare paraneoplastic phenomenon, has occasionally been reported in neuroblastoma.^[14]^ Orbital metastases typically present with periorbital ecchymoses (“raccoon eyes”) and proptosis.^[15]^ In contrast, this case was distinguished by severe headache and acute bilateral blindness, representing an atypical craniofacial manifestation. Overall, this case highlights the spectrum of neuroblastoma presentations and the potential for unusual craniofacial involvement.^[16]^

Noninvasive imaging for neuroblastoma includes ultrasound [US], CT, and MRI.^[17]^ In this patient, abdominal US was not done as a first step which led to a late diagnosis and a higher tumor stage. This shows the importance of US in any abdominal pain. After developing the blindness, an abdominal US and contrast-enhanced CT identified the suprarenal mass. Cranial CT and MRI revealed metastatic lesions in the optic chiasm, sphenoid, and ethmoid regions. Biopsy of the primary mass confirmed stage 4 neuroblastoma (International Neuroblastoma Staging System/stage M, International Neuroblastoma Risk Group Staging System).^[18]^

Standard therapy for high-risk neuroblastoma comprises induction chemotherapy, surgical resection, consolidation with high-dose chemotherapy followed by autologous stem-cell transplantation, radiotherapy, and post-consolidation therapy with isotretinoin and anti-GD2 immunotherapy.^[19]^ In our patient, induction chemotherapy was initiated according to the St. Jude protocol, followed by 90% debulking of the adrenal mass.

Postoperative chemotherapy was continued, and subsequent CT imaging demonstrated regression of metastatic lesions. Autologous stem-cell transplant is planned as consolidation therapy to address marrow infiltration. Radiotherapy and anti-GD2 immunotherapy were not administered due to limitations in availability.

High-risk neuroblastoma carries substantial morbidity from both disease progression and therapy.^[20]^ In this case, intracranial metastasis resulted in bilateral blindness. Tumor involvement of regional lymph nodes and encasement of major vessels further increases the risk of vascular injury during resection.^[21]^ Multimodal therapy adds significant burden, with myelosuppression, infection, hemorrhage, and chemotherapy-related adverse reactions being common.^[22]^ Despite aggressive regimens including multiagent chemotherapy and high-dose chemotherapy with autologous stem-cell rescue, most patients relapse or develop refractory disease, while the few survivors often face long-term sequelae such as hearing loss, restricted growth, diminished fertility, and later-onset malignancies.^[20]^

## 6. Limitations

This case report has several limitations. First, an abdominal ultrasound was not performed at the initial presentation due to limited diagnostic resources in the patient’s region, which contributed to a delay in identifying the underlying pathology. Second, follow-up imaging with CT and MRI was performed only once because of financial constraints, restricting full assessment of the patient’s radiologic response over time. Third, although neuroblastoma frequently involves genetic and molecular abnormalities that influence prognosis and risk stratification, comprehensive genetic testing could not be performed due to restricted availability of advanced diagnostic facilities in Syria. Despite these limitations, this case provides valuable insight into a rare intracranial metastatic presentation of neuroblastoma and reflects the diagnostic challenges encountered in resource-limited settings.

## 7. Conclusion

This case underscores the clinical complexity and aggressive nature of high-risk neuroblastoma, particularly when presenting with atypical manifestations such as bilateral blindness due to intracranial metastases. The diagnostic delay stemming from the absence of early abdominal imaging highlights the critical importance of thorough evaluation in pediatric patients presenting with nonspecific symptoms like abdominal pain and pallor. Despite adherence to established treatment protocols, including surgical debulking and chemotherapy, the irreversible visual impairment in this patient illustrates the devastating consequences of advanced disease. In resource-limited settings, where access to comprehensive imaging and targeted therapies may be constrained, early recognition and prompt intervention remain essential to improving outcomes. This report contributes to the growing body of literature on rare neuroblastoma presentations and reinforces the need for heightened clinical vigilance, multidisciplinary management, and equitable access to care.

## Author contributions

**Resources:** Sultaneh Haddad.

**Supervision:** Heba Amer, Sultaneh Haddad.

**Writing** – **original draft:** Saffaa Ahmed Hersi, Ismail Fawaz Al Malla, Valentina Obaid, Buseyna Dahik, Razan Abbas.

**Writing** – **review & editing:** Heba Amer.
